# Design of Mixed-Mode Analog PID Controller with CFOAs

**DOI:** 10.3390/s24103125

**Published:** 2024-05-14

**Authors:** Natchanai Roongmuanpha, Jetsdaporn Satansup, Tattaya Pukkalanun, Worapong Tangsrirat

**Affiliations:** 1 School of Engineering, King Mongkut’s Institute of Technology Ladkrabang (KMITL), Bangkok 10520, Thailand; natchanai.ro@kmitl.ac.th (N.R.); worapong.ta@kmitl.ac.th (W.T.); 2 Faculty of Engineering, Rajamangala University of Technology Rattanakosin (RMUTR), Nakhon Pathom 73170, Thailand; jetsdaporn.s@rmutr.ac.th

**Keywords:** current-feedback operational amplifier (CFOA), proportional-integral-derivative (PID), analog controller, mixed-mode circuits

## Abstract

The design of a mixed-mode proportional-integral-derivative (PID) controller circuit using current-feedback operational amplifiers (CFOAs) as active components is proposed. With the same circuit topology, the proposed configuration of three CFOAs, four resistors, and two capacitors is capable of performing the PID controller in each of the following four modes: voltage mode, trans-admittance mode, current mode, and trans-impedance mode. Numerous mathematical analyses are conducted to determine the controller’s performance under both ideal and non-ideal conditions. Additionally, the mixed-mode second-order lowpass filter is suggested and also used to examine the workability of the proposed mixed-mode PID controller in a feedback control structure. The proposed PID controller is implemented with the commercially available IC-type CFOA AD844, and the simulation results are presented to illustrate the functionality of the controller and its closed-loop control system. According to the findings, the total power consumption of the proposed PID controller is 0.348 W, with symmetrical supply voltages of ±9 V. It also has a temperature variation of less than 0.2% over the AD844’s usable range. Monte Carlo statistical analysis results revealed that the gain responses of the controller exhibited a deviation of no more than 7.72% from the theoretical value. The controlled filter in a closed-loop control system has a 43% faster rise time and peak time than the uncontrolled filter in all four modes of operation. It also has a steady-state error less than 0.2 mV for voltage responses and 0.72 µA for current responses.

## 1. Introduction

Proportional-integral-derivative (PID) controllers are the most significant control components used in numerous industrial processes [1]. It is estimated that over 90% of all dynamical control systems utilize a PID controller [2]. With its three-term functionality involving proportional, integral, and derivative actions, the PID controller handles the treatment of transient and steady-state responses and adjusts the level of system stability, which are effective solutions for a wide range of real-world control problems [3]. It also offers simplicity, robustness, wide applicability, and simple parameter tuning. As a result, the prevalence of PID control has greatly increased.

A literature review reveals that the PID controller implementation includes a wide variety of designs based on the use of different active elements [4,5,6,7,8,9,10,11,12,13,14,15,16,17,18,19]. In [4], voltage-feedback operational amplifiers (OAs) are extensively used to implement conventional voltage-mode (VM) PID controllers. The realized controller, however, requires a significant number of active and passive components. The constant gain bandwidth product and low slew rate of the OA also limit its response time. To overcome these limitations, some current-mode (CM) active components, such as the operational transconductance amplifier (OTA), current differencing buffered amplifier (CDBA), operational transresistance amplifier (OTRA), second-generation current conveyor (CCII), voltage differencing current conveyor (VDCC), and current-feedback operational amplifier (CFOA), are suggested for PID controller implementations. The OTA-based PID controller in [5] uses two grounded capacitors and eight OTAs. It supplies the output voltage signal at the high-impedance terminal, which is incompatible with cascading in VM. The PID controller designed with CDBAs requires four active and ten passive components and lacks high-input impedance [6]. In [7], the VM PID controller circuit is constructed with two OTRAs, four floating resistors, and three floating capacitors, but it does not have both high input and low output impedances. Based on CCIIs, the PID controller circuits are proposed in [8,9,10]. The works of [8,9] present two distinct configurations for VM and CM operations, while [10] only discusses CM operation. However, none of the CCII-based VM PID designs have low output impedance, and neither of the CM PID designs have low input impedance. As described in [11], a single VDCC-based VM PID controller circuit with a single input and two output terminals is realized with four resistors and two capacitors. It can simultaneously implement non-inverting and inverting control signals. However, the configuration does not fully utilize the differential input property of the VDCC because one of the differential inputs is not employed. This could be the result of input noise injection. Also, a recent VM PID controller using a single active component was reported in [12], but its limitations are the same as those in [11]. Three DDCCs and five passive elements are used in the construction of VM PID controllers [13]. At the inputs of all DDCCs, the high-input impedance and capability of arithmetic operations are not fully utilized. Some earlier works do not exhibit high-input and low-output impedances for VM [14,15] or low-input and high-output impedances for CM [16]. In addition, all of the proposed PID controllers in [4,5,6,7,8,9,10,11,12,13,14,15,16] are capable of operating in either VM or CM. In real-world process control applications, mixed-signal processing PID controllers are required to interact between CM and VM circuits. To satisfy this requirement, the trans-admittance-mode (TAM) and trans-impedance-mode (TIM) PID controller circuits are also used to interface between CM and VM units without any distortion. Only one of those controllers suggests a transconductor-capacitor-based mixed-mode PID design [17]. The active blocks used in the design are not commercially available. Note that implementing the controller with commercially available integrated circuit (IC) elements is advantageous from both a practical and a simplicity aspect. Consequently, the PID controller based on a single commercially available IC CFOA, two resistors, and two grounded capacitors was described in [18]. The design focuses solely on VM operation. In a recent work [19], two CM PID controllers were introduced, each comprising two CFOAs and four passive components. These controllers can be modified to work as mixed-mode PID controllers, capable of operating in all four possible modes. However, the modified controllers lack a high-impedance input feature, causing loading effects with the prior stage for the VM and TAM controllers. Therefore, an additional buffer circuit may be required for VM and TAM operations.

This work describes a mixed-mode PID controller circuit that utilizes three CFOAs as active components in addition to four resistors and two capacitors as passive components. The proposed controller has the capability of performing mixed-mode PID control responses, including VM, CM, TAM, and TIM, within a single topology. Table 1 provides a detailed comparison between the proposed circuit and earlier PID controllers [4,5,6,7,8,9,10,11,12,13,14,15,16,17,18,19]. Therefore, the major contributions of this work are as follows:

(1) We introduce the realization of a PID controller that can operate in all four possible modes without modifying its circuit configuration. The proposed controller provides high-input and low-output impedance properties for the voltage signal as well as low-input and high-output impedance properties for the current signal, allowing direct cascading with any VM or CM plant. By using the TAM and TIM operations, one can establish a connection between a voltage signal and any CM plant, and vice versa. For its implementation, all grounded capacitors are required, and there are no element-matching criteria or cancellation constraints.
(2) The design utilizes a readily available IC-type CFOA AD844 as an active component, which is crucial for ensuring simplicity and practicality when applying the proposed controller. Since the PID controller always uses low frequencies, the use of the model parameters of the commercial CFOA makes the simulation result at lower frequencies match the theoretical results with low tolerances. This is due to the fact that parasitic resistance and capacitance have less of an impact than the CFOA that uses MOS-based parameters [19].
(3) Furthermore, a mixed-mode second-order low-pass filter with a grounded capacitor is suggested, which can be used in all modes of operation, in order to assess the effectiveness of the proposed mixed-mode PID controller.

## 2. Proposed Mixed-Mode PID Controller Configuration

**Figure 1:**
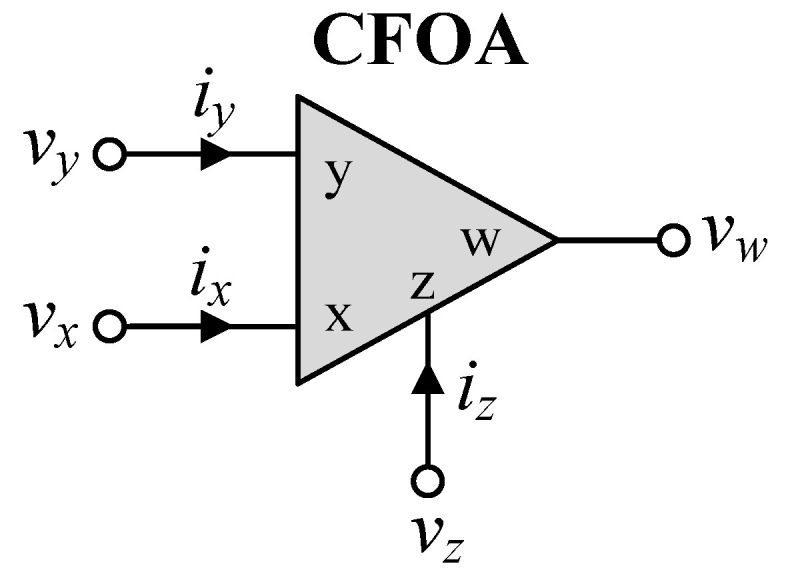
CFOA circuit representation.

The CFOA is a four-terminal active device, represented symbolically in Figure 1. Its ideal characteristic is defined by *i_y_* = 0, *v_x_* = *v_y_*, *i_z_* = *i_x_*, and *v_w_* = *v_z_*. In addition, the characteristics of the CFOA with non-ideal transfer gains can be defined by the following terminal relations:

*i_y_* = 0, *v_x_* = *βv_y_*, *i_z_* = *αi_x_*, and *v_w_* = *γv_z_*, (1)

where *β* = (1 − *ε_β_*), *α* = (1 − *ε_α_*), and *γ* = (1 − *ε_γ_*). Further, *ε_β_* (|*ε_β_*| << 1) is the input-voltage tracking error, *ε_α_* (|*ε_α_*| << 1) is the input-current tracking error, and *ε_γ_* (|*ε_γ_*| << 1) is the output-voltage tracking error. All of the parameters *β*, *α*, and *γ* should ideally equal one.

**Figure 2:**
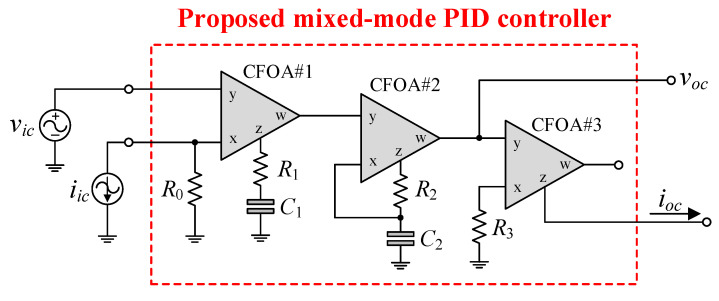
Proposed mixed-mode PID controller configuration using CFOAs.

Figure 2 depicts the configuration of the proposed mixed-mode PID controller, which consists of two input terminals (*v_ic_* and *i_ic_*) and two output terminals (*v_oc_* and *i_oc_*). For the VM signal, the circuit provides high-input and low-output impedance, while for the CM signal, it provides low-input and high-output impedance. By appropriately employing the relevant input signals via *v_ic_* and *i_ic_*, the proposed PID controller can realize all four possible modes of operation, VM, TIM, CM, and TAM, in a single topology. Consequently, it is a mixed-mode PID controller.

The general transfer function of the PID controller can be expressed as:

(2) $$G_{V}(s)=K_{P}+\frac{K_{I}}{s}+sK_{D},$$

where the parameters *K_P_*, *K_I_*, and *K_D_* are the proportional gain, integral gain, and derivative gain of the controller, respectively.

### 2.1. VM and TAM Operations

From Figure 2, if *i_ic_* = 0, one can derive the following generalized transfer function for VM:

(3) $$G_{V}(s)=\frac{v_{oc}}{v_{ic}}=\frac{R_{1}}{R_{0}}\left(1+\frac{R_{2}C_{2}}{2R_{1}C_{1}}\right)+\left(\frac{1}{sR_{0}C_{1}}\right)+\left(\frac{sR_{1}R_{2}C_{2}}{2R_{0}}\right).$$

Comparing Equation (3) to Equation (2), the important gain coefficients of the proposed VM PID controller are obtained as follows:

(4a) $$K_{PV}=\frac{R_{1}}{R_{0}}\left(1+\frac{R_{2}C_{2}}{2R_{1}C_{1}}\right),$$

(4b) $$K_{IV}=\frac{1}{R_{0}C_{1}},$$

and

(4c) $$K_{DV}=\frac{R_{1}R_{2}C_{2}}{2R_{0}}.$$

In Equation (4), the parameters *K_PV_*, *K_IV_*, and *K_DV_* are the gains *K_P_*, *K_I_*, and *K_D_* for VM, respectively.

Similarly, the transfer function of the proposed TAM PID controller is also obtained as:

(5) $$G_{Y}(s)=\frac{i_{oc}}{v_{ic}}=\frac{R_{1}}{R_{0}R_{3}}\left(1+\frac{R_{2}C_{2}}{2R_{1}C_{1}}\right)+\left(\frac{1}{sR_{0}R_{3}C_{1}}\right)+\left(\frac{sR_{1}R_{2}C_{2}}{2R_{0}R_{3}}\right),$$

where

(6a) $$K_{PY}=\frac{R_{1}}{R_{0}R_{3}}\left(1+\frac{R_{2}C_{2}}{2R_{1}C_{1}}\right),$$

(6b) $$K_{IY}=\frac{1}{R_{0}R_{3}C_{1}},$$

and

(6c) $$K_{DY}=\frac{R_{1}R_{2}C_{2}}{2R_{0}R_{3}}.$$

Since the primary objective of this communication is to design an analog PID controller with all four modes of operation in a single configuration, orthogonal adjustment of the control gain parameters *K_PV_*_(*Y*)_, *K_IV_*_(*Y*)_, and *K_DV_*_(*Y*)_ derived from Equations (4) and (6) is not anticipated. However, independent tuning of *K_IV_*_(*Y*)_ and *K_DV_*_(*Y*)_ is possible via *R*_0_ and *C*_2_, respectively, by adjusting *R*_0_, *R*_1_, *C*_1_, and *C*_2_ simultaneously in order that *R*_1_/*R*_0_ and *C*_2_/*C*_1_ remain constant.

### 2.2. CM and TIM Operations

Furthermore, by applying *i_in_* while connecting *v_ic_* to ground (*v_ic_* = 0), the proposed circuit in Figure 2 can be used for CM PID control. As a consequence, the realized transfer function of the CM PID controller is

(7) $$G_{I}(s)=\frac{i_{oc}}{i_{ic}}=\frac{R_{1}}{R_{3}}\left(1+\frac{R_{2}C_{2}}{2R_{1}C_{1}}\right)+\left(\frac{1}{sR_{3}C_{1}}\right)+\left(\frac{sR_{1}R_{2}C_{2}}{2R_{3}}\right).$$

For *R*_0_ = *R*_3_, the gain parameters *K_PI_*, *K_II_*, and *K_DI_* of the CM PID controller in Equation (7) are the exact same as *K_PV_*, *K_IV_*, and *K_DV_* in Equation (4).

It is also observed that the transfer function of the proposed TIM PID controller is found as:

(8) $$G_{Z}(s)=\frac{v_{oc}}{i_{ic}}=\left(R_{1}+\frac{R_{2}C_{2}}{2C_{1}}\right)+\left(\frac{1}{sC_{1}}\right)+\left(\frac{sR_{1}R_{2}C_{2}}{2}\right),$$

where

(9a) $$K_{PZ}=R_{1}+\frac{R_{2}C_{2}}{2C_{1}},$$

(9b) $$K_{IZ}=\frac{1}{C_{1}},$$

and

(9c) $$K_{DZ}=\frac{R_{1}R_{2}C_{2}}{2}.$$

According to Equations (4), (6) and (9), it can be observed that the relative element sensitivities of the control coefficients are low, in that their values are all less than unity, as given below:

(10) $$S_{R_{0}}^{K_{PV}}=S_{R_{0},R_{3}}^{K_{PY}}=S_{R_{3}}^{K_{PI}}=-1,$$

(11) $$S_{R_{1}}^{K_{PV},K_{PY},K_{PI},K_{PZ}}=\frac{1}{\left(1+\frac{R_{2}C_{2}}{2R_{1}C_{1}}\right)}<1,$$

(12) $$S_{R_{2},C_{2}}^{K_{PV},K_{PY},K_{PI},K_{PZ}}=-S_{C_{1}}^{K_{PV},K_{PY},K_{PI},K_{PZ}}=\frac{1}{\left(1+\frac{2R_{1}C_{1}}{R_{2}C_{2}}\right)}<1,$$

(13) $$S_{R_{0},C_{1}}^{K_{IV}}=S_{R_{0},R_{3},C_{1}}^{K_{IY}}=S_{R_{3},C_{1}}^{K_{II}}=S_{C_{1}}^{K_{IZ}}=-1,$$

and

(14) $$S_{R_{0},C_{1}}^{K_{IV}}=S_{R_{0},R_{3},C_{1}}^{K_{IY}}=S_{R_{3},C_{1}}^{K_{II}}=S_{C_{1}}^{K_{IZ}}=-1.$$

## 3. Non-Ideality Effects of CFOA Parasitic Gains

In practice, the CFOA may take into account the non-ideal transfer gains *β*, *α*, and *γ*. According to Equation (1), when *β* ≠ *α* ≠ *γ* ≠ 1, the following are the practical gain parameters of the proposed PID controller in Figure 2.

For VM operation, the control parameters *K_PV_*, *K_IV_*, and *K_DV_* are nonideally obtained as:

(15a) $$K_{PV}=\frac{\beta_{1}\beta_{2}\alpha_{1}\gamma_{1}\gamma_{2}R_{1}}{R_{0}}\left[1+\frac{\alpha_{2}R_{2}C_{2}}{\left(1+\alpha_{2}\right)R_{1}C_{1}}\right],$$

(15b) $$K_{IV}=\frac{\beta_{1}\beta_{2}\alpha_{1}\gamma_{1}\gamma_{2}}{R_{0}C_{1}},$$

and

(15c) $$K_{DV}=\frac{\beta_{1}\beta_{2}\alpha_{1}\alpha_{2}\gamma_{1}\gamma_{2}R_{1}R_{2}C_{2}}{\left(1+\alpha_{2}\right)R_{0}},$$

where the multiplication coefficients *β_i_*, *α_i_*, and *γ_i_* (*i* = 1, 2, 3) denote the non-ideal gains *β*, *α*, and *γ* of the *i*-th CFOA, respectively.

For TAM operation, the non-ideal control parameters *K_PY_*, *K_IY_*, and *K_DY_* can be expressed as:

(16a) $$K_{PY}=\frac{\beta_{1}\beta_{2}\beta_{3}\alpha_{1}\alpha_{3}\gamma_{1}\gamma_{2}R_{1}}{R_{0}R_{3}}\left[1+\frac{\alpha_{2}R_{2}C_{2}}{\left(1+\alpha_{2}\right)R_{1}C_{1}}\right],$$

(16b) $$K_{IY}=\frac{\beta_{1}\beta_{2}\beta_{3}\alpha_{1}\alpha_{3}\gamma_{1}\gamma_{2}}{R_{0}R_{3}C_{1}},$$

and

(16c) $$K_{DY}=\frac{\beta_{1}\beta_{2}\beta_{3}\alpha_{1}\alpha_{2}\alpha_{3}\gamma_{1}\gamma_{2}R_{1}R_{2}C_{2}}{\left(1+\alpha_{2}\right)R_{0}R_{3}}.$$

For CM and TIM operations, the non-ideal control parameters can be determined, respectively, as follows:

(17a) $$K_{PI}=\frac{\beta_{2}\beta_{3}\alpha_{1}\alpha_{3}\gamma_{1}\gamma_{2}R_{1}}{R_{3}}\left[1+\frac{\alpha_{2}R_{2}C_{2}}{\left(1+\alpha_{2}\right)R_{1}C_{1}}\right],$$

(17b) $$K_{II}=\frac{\beta_{2}\beta_{3}\alpha_{1}\alpha_{3}\gamma_{1}\gamma_{2}}{R_{3}C_{1}},$$

(17c) $$K_{DI}=\frac{\beta_{2}\beta_{3}\alpha_{1}\alpha_{2}\alpha_{3}\gamma_{1}\gamma_{2}R_{1}R_{2}C_{2}}{\left(1+\alpha_{2}\right)R_{3}},$$

and

(18a) $$K_{PZ}=\beta_{2}\alpha_{1}\gamma_{1}\gamma_{2}\left[R_{1}+\frac{\alpha_{2}R_{2}C_{2}}{\left(1+\alpha_{2}\right)C_{1}}\right],$$

(18b) $$K_{IZ}=\frac{\beta_{2}\alpha_{1}\gamma_{1}\gamma_{2}}{C_{1}},$$

(18c) $$K_{DZ}=\frac{\beta_{2}\alpha_{1}\alpha_{2}\gamma_{1}\gamma_{2}R_{1}R_{2}C_{2}}{\left(1+\alpha_{2}\right)}.$$

According to Equations (15)–(18), the gain parameters of the proposed PID controller differ slightly owing to the non-ideal transfer gains *β_i_*, *α_i_*, and *γ_i_*. Inspecting these equations implies that all PID control coefficient sensitivities with respect to non-ideal transfer gains of the CFOA are not greater than unity in absolute value.

## 4. Non-Ideality Effects of CFOA Parasitic Impedances

**Figure 3:**
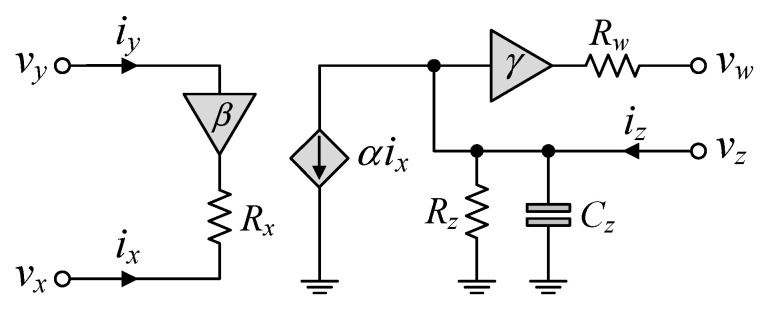
Non-ideal behavior model of the CFOA.

Figure 3 shows the non-ideal behavior model of the practical CFOA, including the typical parasitic impedances. In accordance with this model, *R_x_* and *R_w_* are the low-level parasitic resistances, *R_z_* is the high-level parasitic resistance, and *C_z_* is the parasitic capacitance, associated with the corresponding terminals. For example, the parasitic element values for the commercially available integrated circuit (IC) AD844 CFOA are as follows: *R_x_* = 50 Ω, *R_w_* = 15 Ω, *R_z_* = 3 MΩ, and *C_z_* = 4.5 pF [20].

Considering the dominant parasitic effects of the CFOA on the performance of the proposed mixed-mode PID controller in Figure 2, the following additional assumptions can be defined under the conditions that *β* ≅ *α* ≅ *γ* ≅ 1:

(19) $$\left|R_{1}+\frac{1}{j\omega C_{1}}\right|<<\left|R_{z1}//\frac{1}{j\omega C_{z1}}\right|,$$

and

(20) $$\left|R_{2}+\left(R_{x2}//\frac{1}{j\omega C_{2}}\right)\right|<<\left|R_{z2}//\frac{1}{j\omega C_{z2}}\right|.$$

In practice, the impacts of the parasitic resistances *R_x_*_1_ and *R_x_*_3_ are negligible due to the fact that *R*_0_ >> *R_x_*_1_ and *R*_3_ >> *R_x_*_3_. The expression in Equation (19) can be rewritten as:

(21) $$\sqrt{1+{\left(\omega R_{1}C_{1}\right)}^{2}}<<\frac{\omega R_{z1}C_{1}}{\sqrt{1+{\left(\omega R_{z1}C_{z1}\right)}^{2}}}.$$

When the parasitic capacitance *C_z_*_1_ is minimal, the operating frequency is limited to the following range:

(22) $$f>>\frac{1}{2\pi \left(\sqrt{R_{z1}^{2}-R_{1}^{2}}\right)C_{1}}\Rightarrow f_{1}.$$

Additionally, from Equation (21), if *R_z_*_1_ is negligible, the range of applicable frequencies is as follows:

(23) $$f<<\frac{\sqrt{{\left(\frac{C_{1}}{C_{z1}}\right)}^{2}-1}}{2\pi R_{1}C_{1}}\Rightarrow f_{2}.$$

For instance, if the commercially available IC AD844 CFOA is employed with *R*_1_ = 5 kΩ, and *C*_1_ = 1 nF, the useful operating frequencies found from Equations (22) and (23) are around *f*_1_ ≅ 53 Hz, and *f*_2_ ≅ 7.07 MHz.

Assuming that *R*_2_ >> *R_x_*_2_, Equation (20) can be rearranged as:

(24) $$\sqrt{1+{\left(\omega R_{z2}C_{z2}\right)}^{2}}<<\frac{R_{z2}}{R_{2}}.$$

In the same manner, the practical frequency range in this case is limited to

(25) $$f<<\frac{\sqrt{{\left(\frac{R_{z2}}{R_{2}}\right)}^{2}-1}}{2\pi R_{z2}C_{z2}}\Rightarrow f_{3}.$$

By utilizing *R*_2_ = 5 kΩ, *R_z_*_2_ = 3 MΩ, and *C_z_*_2_ = 4.5 pF, it is possible to determine the frequency location of *f*_3_ ≅ 7.07 MHz.

By combining Equations (22), (23) and (25), the proposed mixed-mode PID controller shown in Figure 2 can be effectively utilized over the following frequency range:

(26) $$max\left\{f_{1}\right\}<<f<<min\left\{f_{2},f_{3}\right\}.$$

## 5. Functional Simulation and Discussion

In order to validate the theoretical analysis presented in the previous section, the proposed mixed-mode PID controller circuit depicted in Figure 2 was investigated using the PSPICE program with model parameters of the commercial CFOA IC-type AD844, available from Analog Devices, Wilmington, MA, USA [20]. All the AD844 ICs were biased with symmetrical power supplies of ±9 V. The passive components for the controller were set as: *R*_0_ = 1 kΩ, *R*_1_ = *R*_2_ = *R*_3_ = 5 kΩ, and *C*_1_ = *C*_2_ = 1 nF. For the specified component values, the controller parameters were calculated as follows:

- *K_PV_* = 7.5, *K_IV_* = 1 Ms^−1^, and *K_DV_* = 12.5 μs for VM;
- *K_PY_* = 1.5 m, *K_IY_* = 200 s^−1^, and *K_DY_* = 2.5 ns for TAM;
- *K_PI_* = 1.5, *K_II_* = 0.2 Ms^−1^, and *K_DI_* = 2.5 μs for CM;
- *K_PZ_* = 7.5 k, *K_IZ_* = 1 Gs^−1^, and *K_DZ_* = 12.5 ms for TIM.

**Figure 4:**
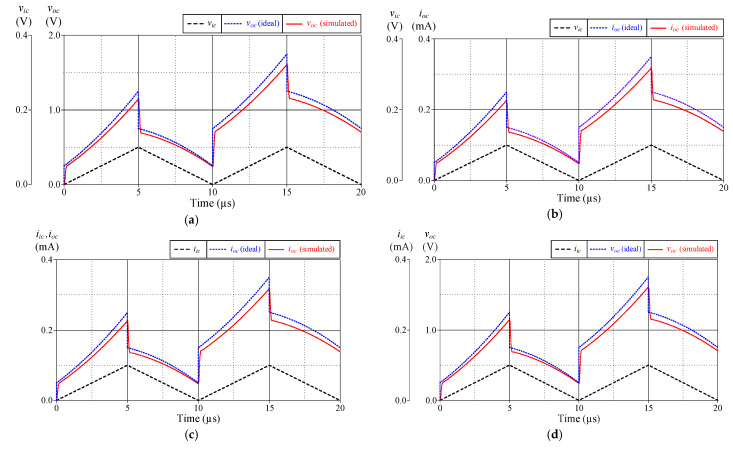
Ideal and simulated time-domain responses of the proposed controller in Figure 2: (**a**) VM; (**b**) TAM; (**c**) CM; (**d**) TIM.

Figure 4 illustrates the time-domain simulation responses of the VM, TAM, CM, and TIM PID controllers in comparison to the ideal responses. As shown in Figure 4, the controller was applied with a 100-kHz triangular input signal with 100 mV peak amplitude for VM and TAM and 100 μA peak amplitude for CM and TIM.

**Figure 5:**
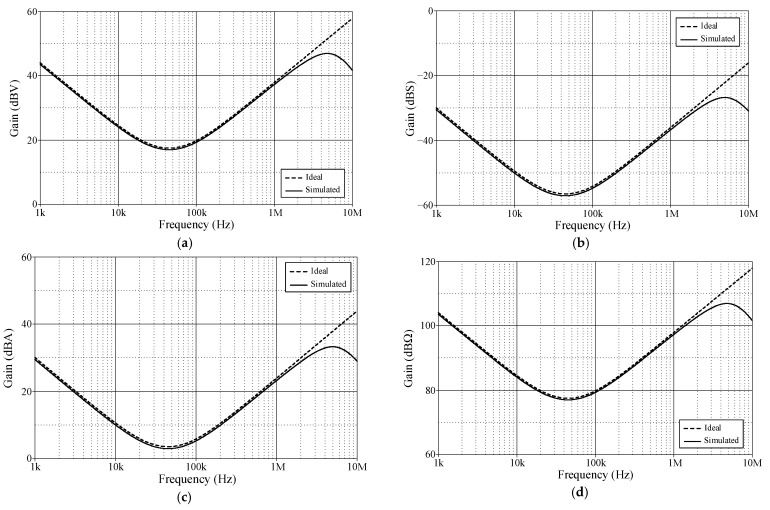
Ideal and simulated frequency-domain responses of the proposed controller in Figure 2: (**a**) VM; (**b**) TAM; (**c**) CM; (**d**) TIM.

Figure 5 additionally illustrates the ideal and simulated frequency-domain characteristics of the proposed mixed-mode PID controller with the exact same components. It is evident from these responses that the gain-frequency limitation of the controller occurs predominantly at more than 5 MHz, with an error of no more than 9.56%. This phenomenon can be attributed to the dominant pole frequencies of parasitic impedances, as expected in the previous section. According to the simulation results, the total power consumption of the controller, comprising static and dynamic power losses, is estimated to be 0.348 W.

**Figure 6:**
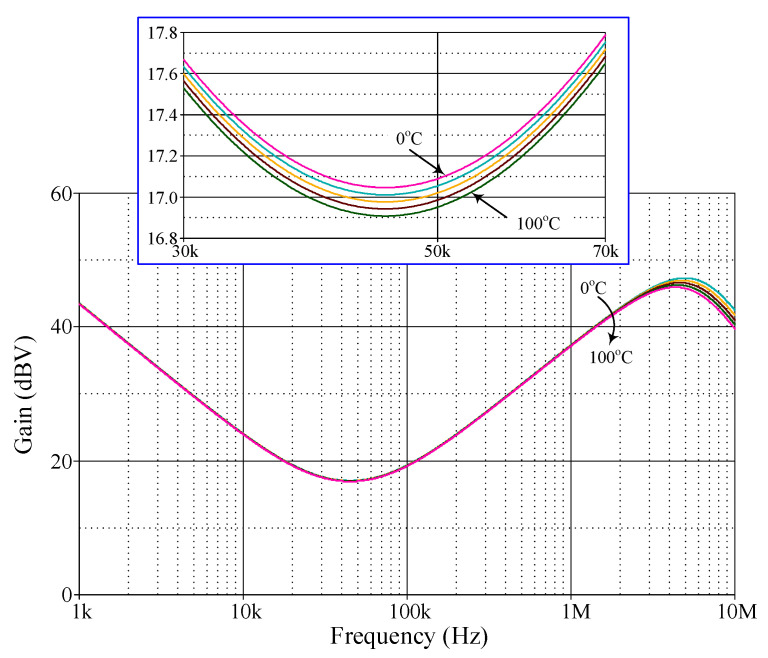
Simulated frequency responses of the proposed VM PID controller with ambient temperature variation.

A further analysis was conducted on the gain response variation in the proposed VM PID controller with respect to ambient temperature. The temperature analysis was performed at the following temperatures: *T* = 0 °C, 25 °C, 50 °C, 75 °C, and 100 °C. Figure 6 illustrates the simulation results of the analysis of ambient temperature, while Table 2 presents the controller gain values for various temperatures. Based on the data given in Table 2, the controller gain change with respect to temperature variation (ΔdBV/Δ*T*) is determined to be 0.137%, 0.138%, and 0.179% at *f* = 10 kHz, 100 kHz, and 1 MHz, respectively.

**Figure 7:**
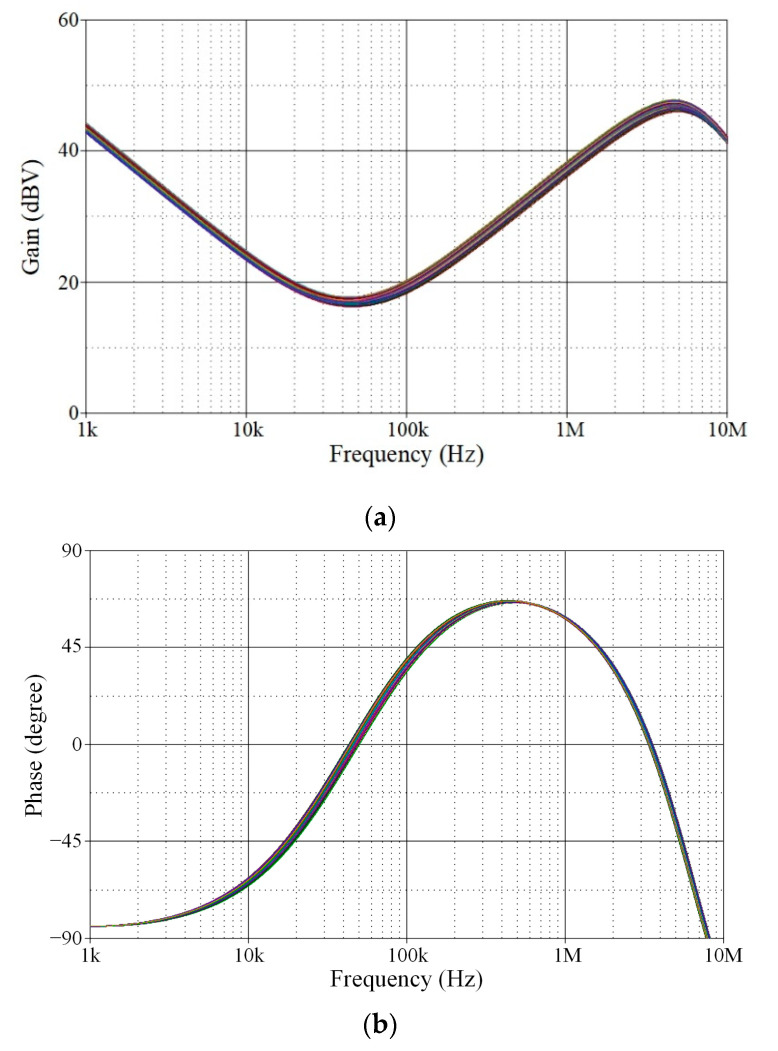
Monte Carlo statistical analysis results of the proposed VM PID controller: (**a**) gain response; (**b**) phase response.

Additionally, Monte Carlo statistical analysis has been performed to demonstrate the robustness of the proposed controller. The analysis was conducted using 200 simulation runs in which the resistor and capacitor values were subject to a 5% Gaussian deviation. The Monte Carlo analysis results are shown in Figure 7, in which the gain responses of the proposed controller deviated from the theoretical value by less than 7.72%. The results indicate that a change in the passive component has no significant effect on the phase or gain responses of the controller.

**Figure 8:**
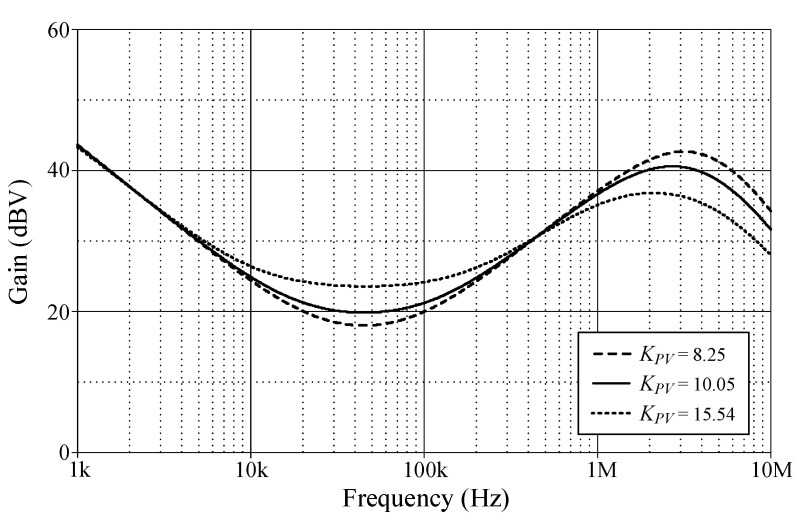
Gain-frequency responses of the proposed VM PID controller when adjusting *K_PV_* while keeping *K_IV_* and *K_DV_* constant.

**Figure 9:**
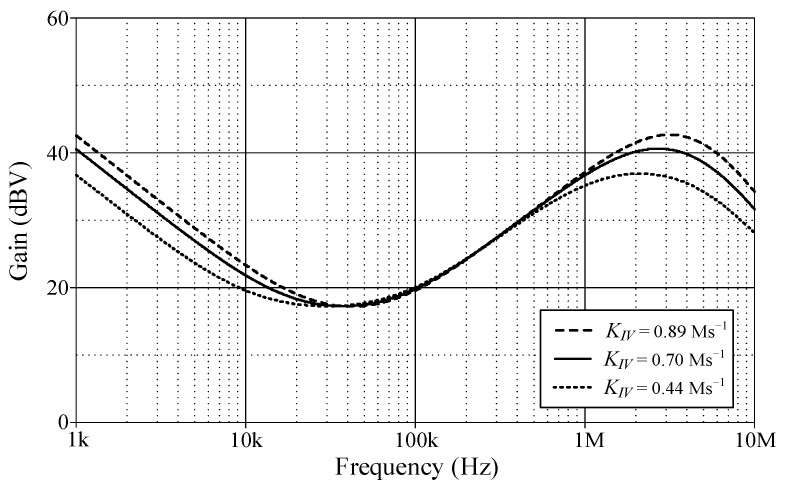
Gain-frequency responses of the proposed VM PID controller when adjusting *K_IV_* while keeping *K_PV_* and *K_DV_* constant.

**Figure 10:**
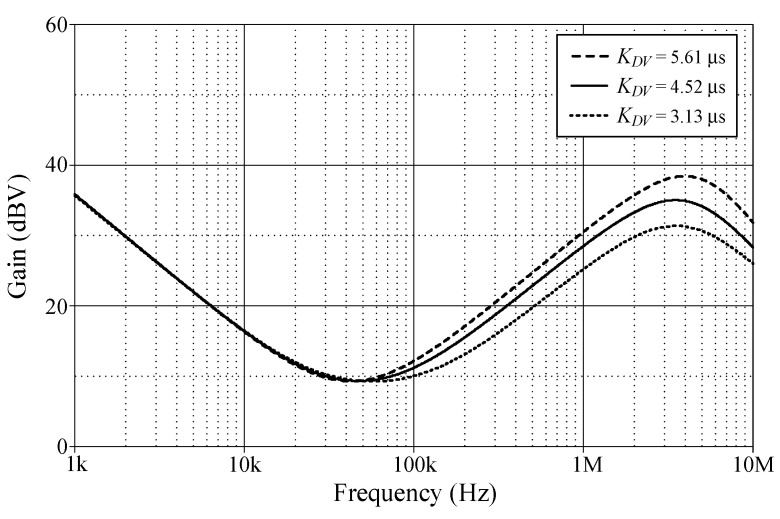
Gain-frequency responses of the proposed VM PID controller when adjusting *K_DV_* while keeping *K_PV_* and *K_IV_* constant.

In order to evaluate the tuning performance, the simulations have been carried out by varying the coefficients *K_PV_*, *K_IV_*, and *K_DV_* of the VM controller. For our first tuning example, the values of various controller components for the variation in controller coefficient *K_PV_*, while holding *K_IV_* and *K_DV_* constants, are given in Table 3. As evident in Figure 8, the parameter *K_PV_* influences the entire operational range, as it appears from the gain response of the controller. The variations in the *K_IV_* and *K_DV_* values resulting from the use of different component values are also provided in Table 4 and Table 5. For the specified set parameters, the gain responses of the VM PID controller with tuning *K_IV_* and *K_DV_* are illustrated in Figure 9 and Figure 10, respectively.

## 6. Performance Verification with Closed-Loop Control Implementation

**Figure 11:**
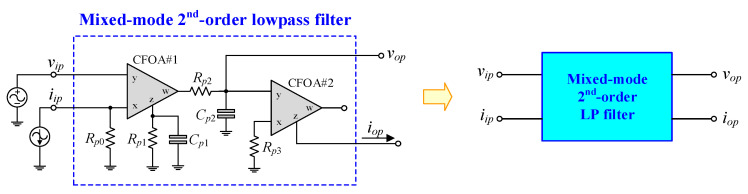
Suggested mixed-mode low-pass filter.

In order to assess the effectiveness of the proposed mixed-mode PID controller in Figure 2, the mixed-mode second-order lowpass (LP) filter depicted in Figure 11 is suggested as a plant for implementing a closed-loop control system. The suggested LP filter can be realized for all four-mode LP filters with the following transfer functions:

(27) $$\;\mathrm{VM}:\;\;\;\;\;\;\;\;\;\;\;\;\;\;\;\;\;\;\;\;\;\;\;\;\;\;\;\;\;\;T_{V}(s)=\frac{v_{op}}{v_{ip}}=\frac{\left(\frac{1}{R_{p0}R_{p2}C_{p1}C_{p2}}\right)}{D(s)},$$

(28) $$\;\mathrm{TAM}:\;\;\;\;\;\;\;\;\;\;\;\;\;\;\;\;\;\;\;\;\;\;\;\;\;\;\;\;\;\;T_{Y}(s)=\frac{i_{op}}{v_{ip}}=\frac{\left(\frac{1}{R_{p0}R_{p2}R_{p3}C_{p1}C_{p2}}\right)}{D(s)},$$

(29) $$\;\mathrm{CM}:\;\;\;\;\;\;\;\;\;\;\;\;\;\;\;\;\;\;\;\;\;\;\;\;\;\;\;\;\;\;T_{I}(s)=\frac{i_{op}}{i_{ip}}=\frac{\left(\frac{1}{R_{p2}R_{p3}C_{p1}C_{p2}}\right)}{D(s)},$$

(30) $$\;\mathrm{TIM}:\;\;\;\;\;\;\;\;\;\;\;\;\;\;\;\;\;\;\;\;\;\;\;\;\;\;\;\;\;\;T_{Z}(s)=\frac{v_{op}}{i_{ip}}=\frac{\left(\frac{1}{R_{p2}C_{p1}C_{p2}}\right)}{D(s)},$$

where

(31) $$D(s)=s^{2}+s\left(\frac{1}{R_{p1}C_{p1}}+\frac{1}{R_{p2}C_{p2}}\right)+\left(\frac{1}{R_{p1}R_{p2}C_{p1}C_{p2}}\right).$$

From Equations (27)–(31), the natural angular frequency (*ω_n_*) and the quality factor (*Q*) for the filter are respectively obtained as:

(32) $$\omega_{n}=2\pi f_{n}=\frac{1}{\sqrt{R_{p1}R_{p2}C_{p1}C_{p2}}},$$

and

(33) $$Q=\frac{\sqrt{R_{p1}R_{p2}C_{p1}C_{p2}}}{R_{p1}C_{p1}+R_{p2}C_{p2}}$$

**Figure 12:**
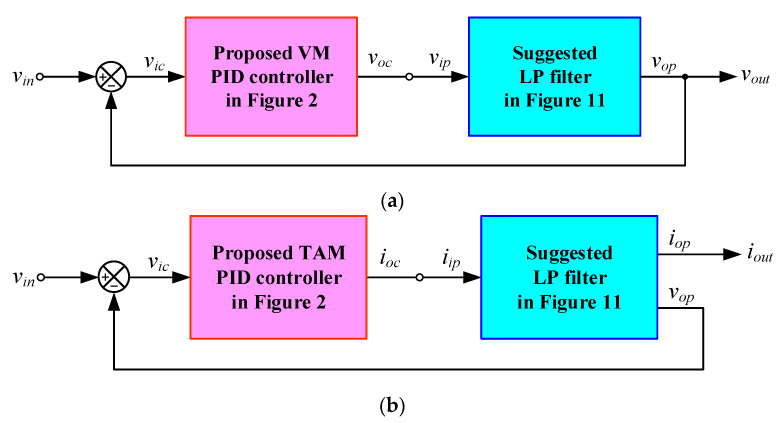

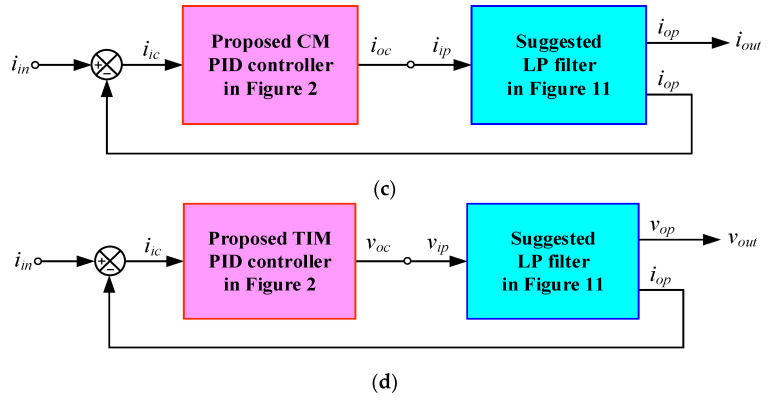
Closed-loop control system implementation: (**a**) VM; (**b**) TAM; (**c**) CM; (**d**) TIM.

The implemented closed-loop systems, as depicted in Figure 12, utilize the mixed-mode PID controller proposed in Figure 2 and the filter plant suggested in Figure 11. The configurations depicted in Figure 12a–d were constructed for the performance assessment of VM, TAM, CM, and TIM controllers, respectively. The component values for the filters are as follows: *R_p_*_0_ = *R_p_*_1_ = *R_p_*_2_ = *R_p_*_3_ = 1 kΩ, and *C_p_*_1_ = *C_p_*_2_ = 1 nF; thus, *f_n_* = 159 kHz and *Q* = 0.5 are obtained. All implemented controllers utilized *R*_1_ = *R*_3_ = 5 kΩ and *C*_1_ = *C*_2_ = 200 pF.

**Figure 13:**
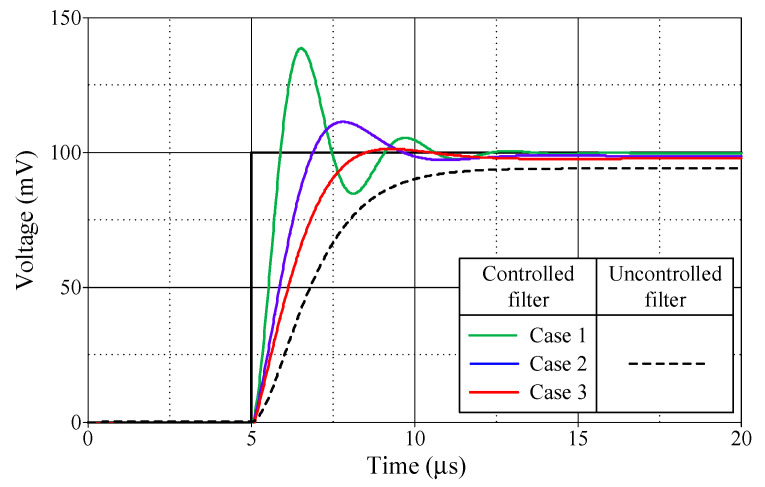
Step-responses of the uncontrolled and controlled filters in Figure 12a.

**Figure 14:**
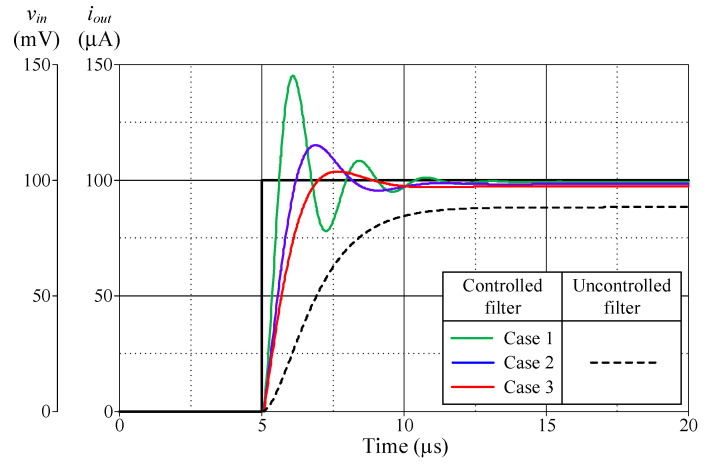
Step-responses of the uncontrolled and controlled filters in Figure 12b.

**Figure 15:**
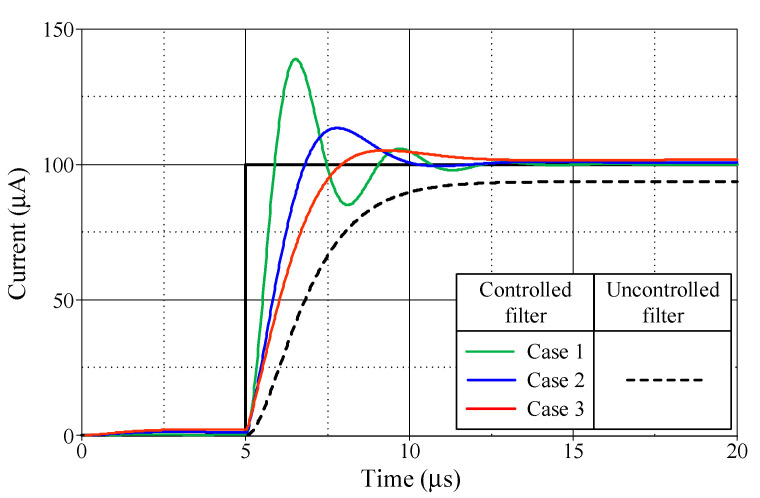
Step-responses of the uncontrolled and controlled filters in Figure 12c.

**Figure 16:**
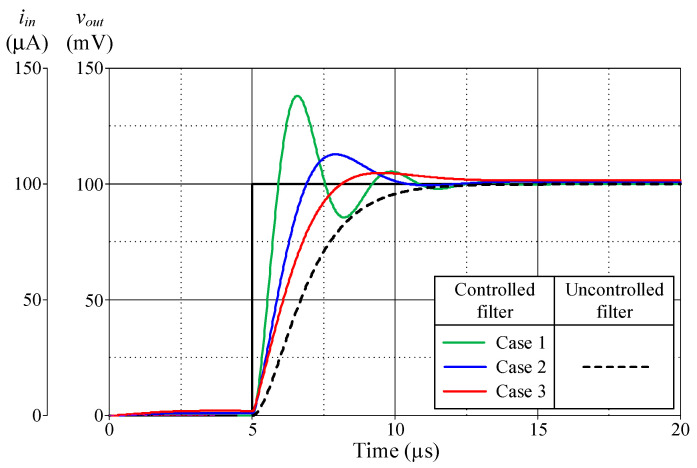
Step-responses of the uncontrolled and controlled filters in Figure 12d.

Figure 13, Figure 14, Figure 15 and Figure 16 illustrate the step responses of the uncontrolled filter and PID-controlled filter systems for Figure 12a–d, respectively. The controller parameters employed to evaluate the step response, along with the characteristics derived from the responses for each of the four modes, are also documented in Table 6, Table 7, Table 8 and Table 9. It is evident from the tables that the proposed PID controllers improved the time response of the closed-loop control filter systems, particularly for *t_d_*, *t_r_*, *t_p_*, and *t_s_*. The times *t_r_* and *t_p_* of the controlled filter in a closed-loop control system were 43% faster than those of the uncontrolled filter in all four modes of operation. It also had a steady-state error of less than 0.2 mV for voltage responses and 0.72 µA for current responses. Additionally, the controlled filters entered the steady-state faster than the uncontrolled filters and tracked the step-input with a reduced steady-state error.

## 7. Conclusions

This work presents a tunable mixed-mode PID controller implemented with commercially available integrated circuits (ICs) and current-feedback operational amplifiers (CFOAs). The presented PID controller circuit employs three CFOAs, four resistors, and two capacitors. All four operational-mode PID controllers, namely VM, TAM, CM, and TIM, can be performed with a single proposed circuit topology. The important parameters of the proposed PID controllers, namely, *K_P_*, *K_I_*, and *K_D_*, are modifiable as desired. Since the proposed controller is designed using only off-the-shelf ICs together with some passive components, it has advantages in terms of practicality and simplicity. Analyses of non-ideal transfer gain and parasitic effects on the controller performance have also been examined in detail. In addition, to evaluate the practical applicability of the proposed mixed-mode PID controller, the mixed-mode second-order lowpass filter is designed to be a testing plant for a mixed-mode closed-loop control system. A simulation study demonstrates the performance of the circuit.

## Figures and Tables

**Table 1:** Comparison features of the proposed controller circuit with the earlier PID controllers [4,5,6,7,8,9,10,11,12,13,14,15,16,17,18,19].

Ref.	Active Component	Passive Component	Mixed-Mode Operation	Operating Modes				Technology	Supply Voltage (V)
				VM	CM	TAM	TIM		
[4]	OA = 4	R = 8, C = 2	no	yes	no	no	no	NA	NA
[5]	OTA = 8	C = 2	no	yes	no	no	no	0.8-μm AMS	±5, (−2~4)
[6]	CDBA = 4	R = 8, C = 2	no	yes	no	no	no	0.8-μm AMS	±2.5, ±1
[7]	OTRA = 2	R = 4, C = 3	no	yes	no	no	no	0.18-μm MOSIS	±1.5
[8]	CCII = 2	R = 4, C = 2	no	yes	yes	no	no	AD844	NA
[9]	CCII = 1, DO-CCII = 1	R = 3, C = 2	no	yes	yes	no	no	0.35-μm TSMC	±1.5, +0.5
[10]	DO-CCII = 1	R = 2, C = 2	no	no	yes	no	no	0.13-μm CMOS	±1, +0.4
[11]	VDCC = 1	R = 4, C = 2	no	yes	no	no	no	0.18-μm CMOS	±0.9
[12]	DVCCTA = 1	R = 3, C = 2	no	yes	no	no	no	0.25-μm TSMC	±1.5, −1
[13]	DDCC = 3	R = 3, C = 2	no	yes	no	no	no	0.13-μm IBM	±0.75, +0.37
[14]	ZC-CFTA = 1	R = 2, C = 2	no	yes	no	no	no	0.35-μm BiCMOS	±1
[15]	CCTA = 1	R = 2, C = 2	no	yes	no	no	no	0.35-μm TSMC	±1.5
[16]	CFOA = 2	R = 3, C = 2 for VM, R = 5, C = 2 for CM	no	yes	yes	no	no	AD844	±12
[17]	Transconductor = 6	C = 2	yes	yes	yes	yes	yes	0.18-μm TSMC	±0.9
[18]	CFOA = 1	R = 2, C = 2	no	yes	no	no	no	0.18-μm TSMC	±2
[19]	CFOA = 2	R = 2, C = 2 for CM (Figure 3)	no	no	yes	no	no	0.18-μm TSMC	±2
		R = 4, C = 2 for MM, (Figure 4)	yes	yes	yes	yes	yes		
Proposed controller	CFOA = 3	R = 4, C = 2	yes	yes	yes	yes	yes	AD844	±9

Abbreviations: R = Resistor, C = Capacitor, NA = Not Available, MM = mixed mode, OA = operational amplifier, OTA = operational transconductance amplifier, CDBA = current differencing buffered amplifier, OTRA = operational transresistance amplifier, CCII = second-generation current conveyor, DO-CCII = dual-output CCII, VDCC = voltage differencing current conveyor, CCTA = current conveyor transconductance amplifier, DVCCTA = differential voltage CCTA, DDCC = differential difference current conveyor, ZC-CFTA = z-copy current follower transconductance amplifier, CFOA = current feedback operational amplifier.

**Table 2:** Temperature dependence of the gain value for the proposed VM PID controller.

Temperature (°C)	Controller VM Gain (dBV)		
	*f* = 10 kHz	*f* = 100 kHz	*f* = 1 MHz
0	24.090	19.350	37.336
25	24.056	19.315	37.293
50	24.021	19.281	37.249
75	23.987	19.246	37.204
100	23.953	19.212	37.157

**Table 3:** Different component values for the variation in controller coefficient *K_PV_*.

*K* *_PV_*	*K_IV_* (Ms^−1^)	*K_DV_* (μs)	*R*_0_ (kΩ)	*R*_1_ = *R*_2_ = *R*_3_ (kΩ)	*C*_1_ (*nF*)	*C*_2_ (*nF*)
8.25	1	12.5	2.5	5	0.40	2.5
10.05			3.5	5	0.29	3.5
15.54			6.0	5	0.17	6.0

**Table 4:** Different component values for the variation in controller coefficient *K_IV_*.

*K_PV_*	*K_IV_* (Ms^−1^)	*K_DV_* (μs)	*R*_0_ (kΩ)	*R*_1_ = *R*_2_ = *R*_3_ (kΩ)	*C*_1_ (nF)	*C*_2_ (nF)
7.5	0.89	12.5	2.5	5	0.45	2.5
	0.70		3.5	5	0.41	3.5
	0.44		6.0	5	0.38	6.0

**Table 5:** Different component values for the variation in controller coefficient *K_DV_*.

*K_PV_*	*K_IV_* (Ms^−1^)	*K_DV_* (μs)	*R*_0_ (kΩ)	*R*_1_ = *R*_2_ = *R*_3_ (kΩ)	*C*_1_ (nF)	*C*_2_ (nF)
3	0.4	5.61	3.5	5	0.72	1.57
		4.52	6	5	0.42	2.17
		3.13	10	5	0.25	2.50

**Table 6:** Controller parameters and the resulting characteristics of the uncontrolled filter and controlled filter systems shown in Figure 12a.

		*R*_0_ = *R*_2_ (kΩ)	*K_PV_*	*K_IV_* (Ms^−1^)	*K_DV_* (μs)	Delay Time, *t_d_* (μs)	Rise Time, *t_r_* (μs)	Peak Time, *t_p_* (μs)	Settling Time, *t_s_* (μs)		Maximum Overshoot, *M_p_* (mV)	Steady- State Error (mV)
									5%	2%		
PID- controlled filter	Case 1	1	5.50	5.00	0.5	5.52	5.89	6.53	9.97	11.52	138.63	0.20
	Case 2	3	2.17	1.67	0.5	5.86	6.88	7.81	9.06	9.52	111.43	1.19
	Case 3	5	1.50	1.00	0.5	6.12	8.50	9.28	7.66	10.66	101.40	2.19
Uncontrolled filter						6.79	11.69	11.69	9.72	10.83	94.19	5.81

**Table 7:** Controller parameters and the resulting characteristics of the uncontrolled filter and controlled filter systems shown in Figure 12b.

		*R*_0_ = *R*_2_ (kΩ)	*K_PY_*	*K_IY_* (Ms^−1^)	*K_DY_* (μs)	Delay Time, *t_d_* (μs)	Rise Time, *t_r_* (μs)	Peak Time, *t_p_* (μs)	Settling Time, *t_s_* (μs)		Maximum Overshoot, *M_p_* (μA)	Steady- State Error (μA)
									5%	2%		
PID- controlled filter	Case 1	1	11	10	1	5.36	5.61	6.09	8.83	10.04	145.23	0.72
	Case 2	3	4.33	3.33	1	5.56	6.21	6.89	7.92	9.64	115.20	1.68
	Case 3	5	3	2	1	5.69	6.98	7.68	8.28	9.09	103.76	2.63
Uncontrolled filter						6.92	14.30	14.30	9.64	10.76	88.36	11.64

**Table 8:** Controller parameters and the resulting characteristics of the uncontrolled filter and controlled filter systems shown in Figure 12c.

		*R*_0_ = *R*_2_ (kΩ)	*K_PI_*	*K_II_* (Ms^−1^)	*K_DI_* (μs)	Delay Time, *t_d_* (μs)	Rise Time, *t_r_* (μs)	Peak Time, *t_p_* (μs)	Settling Time, *t_s_* (μs)		Maximum Overshoot, *M_p_* (μA)	Steady- State Error (μA)
									5%	2%		
PID- controlled filter	Case 1	1	1.1	1	0.1	5.52	5.89	6.53	9.99	11.45	138.92	0.10
	Case 2	3	1.3	1	0.3	5.83	6.81	7.80	9.06	9.51	113.53	0.90
	Case 3	5	1.5	1	0.5	6.03	7.98	9.26	7.66	10.66	105.29	1.71
Uncontrolled filter						6.81	11.63	11.63	9.72	10.83	93.74	6.26

**Table 9:** Controller parameters and the resulting characteristics of the uncontrolled filter and controlled filter systems shown in Figure 12d.

		*R*_0_ = *R*_2_ (kΩ)	*K_PZ_*	*K_IZ_* (Ms^−1^)	*K_DZ_* (μs)	Delay time, *t_d_* (μs)	Rise Time, *t_r_* (μs)	Peak Time, *t_p_* (μs)	Settling Time, *t_s_* (μs)		Maximum Overshoot, *M_p_* (mV)	Steady- State Error (mV)
									5%	2%		
PID- controlled filter	Case 1	1	5.50	5	0.5	5.54	5.92	6.59	10.06	11.62	138.03	0.001
	Case 2	3	6.5	5	1.5	5.87	6.91	7.91	9.19	9.67	112.82	0.782
	Case 3	5	7.5	5	2.5	6.09	8.13	9.51	7.81	10.79	104.79	1.579
Uncontrolled filter						6.70	11.81	11.81	9.79	10.91	99.92	0.085

## Data Availability

Data are contained within the article.
